# Draft Genome Sequence of *Monaibacterium marinum* C7^T^, Isolated from Seawater from the Menai Straits, Wales, United Kingdom

**DOI:** 10.1128/genomeA.01444-17

**Published:** 2018-02-01

**Authors:** Tatyana N. Chernikova, Nikos Kyrpides, Rafael Bargiela, Tanja Woyke, Nicole Shapiro, William B. Whitman, Peter N. Golyshin

**Affiliations:** a School of the Biological Sciences, Bangor University, Bangor, Gwynedd, United Kingdom; b Department of Microbiology, University of Georgia, Athens, Georgia, USA; c DOE, Joint Genome Institute, Walnut Creek, California, USA

## Abstract

Here, we report the draft genome sequence of *Monaibacterium marinum* C7^T^, a strain that represents a new member of the *Roseobacter* clade of the family *Rhodobacteraceae* (*Alphaproteobacteria*). The genome size of *Monaibacterium marinum* C7^T^ is 3.7 Mb (3,734,267 bp), with a G+C content of 58.86%.

## GENOME ANNOUNCEMENT

The *Roseobacter* clade within the *Rhodobacteraceae* (*Alphaproteobacteria*) represents a distinct and diverse group of microorganisms that constitute up to 25% of the coastal marine bacteria (1, 2). The study of the genomic features of members of this clade provides an insight into their ecology, physiology, niche specificity, and adaptation to the environmental stimuli.

The new strain *Monaibacterium marinum* C7^T^ was isolated from a seawater sample taken from Menai Straits (Wales, UK) and represents a distinct lineage within the *Roseobacter* clade of the family *Rhodobacteraceae* within the *Alphaproteobacteria* (3). Strain C7^T^ is an aerobic Gram-negative mesophilic nonmotile short-rod-shaped bacterium that can grow under microaerophilic conditions. It grows on ONR7a medium (4) in the range of NaCl concentrations of 0 to 9% (wt/vol), at temperatures between 4 and 31°C, and in pH range 5.5 to 9.0 with the optimum pH of 7.5.

The DNA was isolated using Meta-G-Nome DNA isolation kit (Epicentre Biotechnologies), according to the manufacturer’s instructions.

The genome of *Monaibacterium marinum* C7^T^ was sequenced in the framework of the project Genomic Encyclopedia of Bacteria and Archaea, phase III, at the Department of Energy (DOE) Joint Genome Institute (JGI) using Illumina technology (5). Sequencing of a standard shotgun library with 300-bp inserts using the Illumina 9 HiSeq 2000 1-Tb platform generated 6,934,098 reads totaling 1,040.1 Mbp of sequence. Known Illumina artifacts were removed, and PhiX sequencing was done by filtering all raw Illumina sequence data using BBDuk (6). Reads with fewer than 51 bp after trimming were discarded from the analysis. The remaining reads were subjected to sequence mapping and masking using BBMAP (6) and BBMask (6), respectively. Assembly was performed using SPAdes version 3.6.2 (7). Annotation was done using the JGI-IMG Annotation Pipeline version 4.14.1.

The final draft assembly contained 27 contigs in 27 scaffolds, totaling 3.735 Mbp in size, and was based on 1,040.1 Mbp of Illumina data with a mapped coverage of 279.2-fold.

The largest contig was 1,171.7 kb. The total number of genes was 3,615, including 57 RNA genes (rRNA, 6; 5S RNA, 4; 16S RNA, 1; 23S RNA, 1; tRNA, 42; other RNA genes 3). Moreover, annotation revealed 3,558 protein-coding genes, with 2,915 having functional predictions. Analysis of the draft genome sequence of *Monaibacterium marinum* C7^T^ using AromaDeg (8) and BLASTP against an in-house database (9), which contains experimentally characterized enzymes involved in the catabolism of aliphatic and aromatic compounds, predicted genes encoding two alkane hydroxylases and two P450 cytochromes (albeit, this strain was not able to grow on aliphatic hydrocarbons of carbon chain length 10 to 20 [3]), one catechol 1,2 dioxygenase for the *ortho*-pathway, and three carboxymuconate cycloisomerases. In addition, the gene inspection using antiSMASH (10) revealed the presence of gene clusters for the biosynthesis of terpenes, homoserine lactones, aryl propylene, and ladderanes.

### Accession number(s).

This draft genome sequence of *Monaibacterium marinum* C7^T^ has been deposited in DDBJ/ENA/GenBank under the accession no. OCTN01000001 to OCTN01000027. The versions described in this paper are the first versions, OCTN01000001.1 to OCTN01000027.1.
